# Animal models of Parkinson’s disease: a guide to selecting the optimal model for your research

**DOI:** 10.1042/NS20210026

**Published:** 2021-12-08

**Authors:** Joana Lama, Yazead Buhidma, Edward J.R. Fletcher, Susan Duty

**Affiliations:** King’s College London, Institute of Psychiatry, Psychology and Neuroscience, Wolfson Centre for Age Related Diseases, Wolfson Wing, Hodgkin Building, Guy’s Campus, London SE1 1UL, U.K.

**Keywords:** Animal models, motor deficits, neuropathology, non-motor symptoms, Parkinson's disease, pathogenesis

## Abstract

Parkinson’s disease (PD) is a complex, multisystem disorder characterised by α-synuclein (SNCA) pathology, degeneration of nigrostriatal dopaminergic neurons, multifactorial pathogenetic mechanisms and expression of a plethora of motor and non-motor symptoms. Animal models of PD have already been instructive in helping us unravel some of these aspects. However, much remains to be discovered, requiring continued interrogation by the research community. In contrast with the situation for many neurological disorders, PD benefits from of a wide range of available animal models (pharmacological, toxin, genetic and α-synuclein) but this makes selection of the optimal one for a given study difficult. This is especially so when a study demands a model that displays a specific combination of features. While many excellent reviews of animal models already exist, this review takes a different approach with the intention of more readily informing this decision-making process. We have considered each feature of PD in turn – aetiology, pathology, pathogenesis, motor dysfunctions and non-motor symptoms (NMS) – highlighting those animal models that replicate each. By compiling easily accessible tables and a summary figure, we aim to provide the reader with a simple, go-to resource for selecting the optimal animal model of PD to suit their research needs.

## Introduction

Parkinson’s disease (PD) is the most rapidly growing neurodegenerative disorder already affecting more than 6.2 million people worldwide, with the number predicted to rise to more than 12 million by 2040 [1]. PD was initially characterised by its motor dysfunction; however, we now know that it is a multisystem disorder which features both motor and non-motor symptoms. These will be elaborated on in the main body of this review, alongside the key pathological features of the disease. At the point of diagnosis, when bradykinesia in combination with a second motor symptom is observed, approx. 50% of dopaminergic neurons in the substantia nigra pars compacta (SNpc) have already degenerated, leading to marked striatal dopamine depletion. Prior to this, in the so-called prodromal stage of PD, a range of non-motor symptoms (NMS) including hyposmia, constipation, mood disorders and rapid eye movement (REM) sleep behaviour disorder are commonly seen. Studies of the prodromal phase may help establish biomarkers to enable earlier diagnosis and thereby facilitate earlier intervention with neuroprotective, disease-modifying therapies when these become available.

Animal models are useful tools for the experimental study of distinctive characteristics and aspects of the disease. While many excellent reviews of animal models of PD have already been published [2–5], these usually focus on each model in turn, presenting a detailed description of the pathology, symptoms and features they offer. In this review we have taken a different approach. We have summarised the journey of Parkinson’s, from pathology and pathogenesis to motor and non-motor symptoms. We focus on the different features of PD, one at a time, and showcase the animal models which recapitulate each. By presenting these also in table and figure format, we aim to compile a convenient, but comprehensive guide of available animal models for researchers to select from when exploring a specific feature of PD, be this a particular pathology (e.g. synucleinopathy) or NMS (e.g. pain). Our ultimate goal is to furnish the reader with a go-to resource; a compendium of current animal models to suit their research needs.

## Animal models of PD: a brief overview

In contrast with the situation for many other neurodegenerative diseases, PD benefits from of a wide range of available animal models, the different classes of which (pharmacological, toxin, genetic and α-synuclein) are briefly summarised below. We have focused here on the mammalian models; readers interested in the various non-mammalian models such as those in *Drosophila melanogaster* or *Caenorhabditis elegans* are directed towards existing reviews [6–8].

The **pharmacological models** of PD were the first ones developed and contributed to an extent in the discovery of symptomatic drugs, such as the gold standard, levodopa (L-DOPA) [9]. Reserpine, an inhibitor of the vesicular monoamine transporters (VMATs), is administered peripherally. When given in a single dose, reserpine induces depletion of all monoamines including noradrenaline and serotonin alongside the critical one, dopamine which renders rodents severely, but transiently, akinetic [10]. More recently, repeated low-dose administration of reserpine has been used to produce a progressive model with additional relevant characteristics for interrogation [11]. It will be interesting to see how well-replicated and widely adopted this particular model becomes in the future. A second, less commonly used pharmacological model is induced by peripheral administration of haloperidol, an antagonist of dopamine D_2_ and, to a lesser extent, D_1_ receptors. Post-treatment, haloperidol induces a transient catalepsy with animals unable to right their posture [12]. Although transient, the pharmacological models are certainly the easiest to generate as they require no specialist stereotaxic equipment, surgical skills, or adapted housing for example, unlike most of the models outlined below.

For more permanent effects, **toxin models** have been developed. These toxins can be broadly subcategorised into neurotoxins (6-hydroxydopamine; 6-OHDA and 1-methyl-4-phenyl-1,2,3,6-tetrahydropyridine; MPTP), pesticides (rotenone, paraquat and permethrin) and endotoxins (lipopolysaccharide; LPS). The mechanism for how each of these toxins cause their degenerative effects is reviewed in detail elsewhere [2]. In brief, when administered intracranially into the nigrostriatal tract (for 6-OHDA and LPS) or systemically (for MPTP, rotenone, paraquat and permethrin), they mostly cause disruption of mitochondrial complexes involved in oxidative phosphorylation, alongside an increase in reactive oxidative species and ultimately nigral cell death. LPS, on the other hand, is thought to induce a PD-like phenotype through enhancing microgliosis and local iron and ferritin levels at the site of injection [23]. Typically, these toxin models induce a rapid loss of dopaminergic cells that gives rise to motor dysfunction and further behavioural deficits.

Despite their frequent use, these commonly used models have yet to fully recapitulate the progressive nature of the disease. However, this situation may change in the future. One toxin model of PD, not elaborated on below because it is not yet so well-established, is the β-sitosterol d-glucoside (BSSG) model. Unlike the above-mentioned toxins, BSSG is a neurotoxin isolated from the cycad plant which, when administered orally to rats 5 days per week for 16 weeks, is reported to cause a progressive nigrostriatal degeneration over the subsequent 4–6 months [13]. This is accompanied by a progressive topographical deposition of pathological α-synuclein aggregates, similar to the pattern of spread reported in humans and the presentation of NMS such as olfactory dysfunction prior to motor impairment and ultimately cognitive impairment [13]. This exciting model, that seems to faithfully reproduce many aspects of the human condition, has recently been replicated using a single intranigral administration of BSSG [14]. When administered by this route, BSSG still induces bilateral progressive pathology and olfactory, motor and cognitive symptoms similar to those seen in the oral model but starting as early as 15 days post-injection. However, in this intracerebral model, olfactory dysfunction occurs concurrently with the start of motor impairment, mirroring less the timing of the clinical situation. Nevertheless, it will be interesting to see whether the BSSG models becomes more widely adopted in the next few years.

With the discovery of several familial forms of PD, researchers have generated animal models that attempt to replicate the disease through genetic mutation. These **genetic models** are numerous and therefore cannot all be afforded the attention they deserve here. Indeed, there are at least 13 different SNCA mutation-based models alone. Accordingly, we have restricted our focus to those models with clear links to both familial and sporadic cases: α-synuclein (SNCA), leucine-rich repeat kinase (LRRK2) and glucocerebrosidase (GBA). The features of the different SNCA models are the subject of a recent review to which the reader is also directed [4].

SNCA has been implicated in the pathology of PD and several groups have studied its spreading, toxicity, misfolding and aggregation [15–17]. These studies have led to the development of non-transgenic **α-synuclein models** that aim to replicate the synucleinopathy seen in PD, taking advantage of its spreading to promote the pathology [18]. Adeno-associated viral vectors (AAVs) have been used for some time to overexpress α-synuclein in the central nervous system of wildtype rodents and non-human primates (NHPs) [19–21]. This model has taught us about the pathophysiology of α-synuclein and the importance of its expression in the severity and development of PD. However, it has proven difficult to standardise between laboratories, with different methods of AAV production and purity, for example. More recently, the pre-formed fibril (PFF) model has started to gain traction. In brief, human, or rodent α-synuclein PFFs are injected once, most often into the striatum, of rats or mice to drive the aggregation of endogenous α-synuclein into Lewy body (LB)-like inclusions over a period of 3–6 months [18,22]. This exciting model replicates several features of PD, which will be discussed further in the main body of this review, but again the issue of standardising the protocol is undergoing active discussion at the current time [23]. In some studies, low-titre injections of AAV–α-synuclein have been combined with subthreshold doses of injected PFFs of the same species. When given into both the SN and ventral tegmental area in rats, this resulted in speeding up the process of aggregate formation and progressive neurodegeneration, alongside an enhanced degree of motor impairment, when compared with either AAV or PFF delivery alone [24]. This model has not been extensively studied, however, so we restrict our description below to the two separate models only.

Having briefly introduced the different classes of mammalian model, we now turn our attention to the key features of PD. Specifically, we provide a concise summary of each feature from aetiology through to NMS and, most importantly, emphasise which animal models are suitable for experimental investigations of that particular feature. Although we include many of the non-motor features associated with prodromal PD, given that investigation of this disease stage in animal models is in its relative infancy, we omit specific reference to it here and instead direct readers seeking insight into animal models of prodromal PD to the recent review by Taguchi et al. [25].

## Modelling aetiology

Although the exact cause of PD in most cases remains unknown – so-called idiopathic PD – and occurs sporadically, certain risk factors are acknowledged, with those of age, environmental toxins and genetics being most well established. These are all, to some extent, accounted for in the available array of animal models of PD.

### Age

Age is certainly one of the biggest risk factors for developing PD [26], yet it is often overlooked when generating the pharmacological or toxin-based models. While the use of older animals will prove more difficult (e.g., for stereotaxic surgery and behavioural tests) and more expensive (to purchase or to age in-house) than using young adults, they would maximise the clinical translatability of outputs from these models given that most cases of PD are diagnosed in older individuals. Other models include ageing as part of their generation, thereby increasing translatability. For example, many transgenic models only display relevant phenotypes when the animals are over 12 months old, while the α-synuclein PFF models also do not exhibit degeneration or motor impairment until 6–9 months after PFF injection.

### Environmental toxins

It is nearly 40 years since the discovery by Langston et al. (1983) that MPTP was linked to nigrostriatal degeneration, following the appearance of characteristic PD symptoms in a group of individuals who injected themselves with MPTP-contaminated ‘heroin’ [27]. From this realisation emerged the idea that PD could be caused by an environmental toxin. Rural living and exposure to pesticides are recognised as risk factors for PD [28] and the latter risk is certainly well replicated by the toxin-based models generated using pesticides (paraquat, rotenone and permethrin).

### Genetics

Approx. 10–15% of PD cases have monogenic forms of the disease associated with mutations in over 20 genes [29]. These rare mutations range from the autosomal recessive, such as PINK1 and Parkin to the autosomal dominant, such as LRRK2 and SNCA. Animal models generated with such familial mutations are helpful for researchers wishing to unravel the consequences of these mutations at a molecular, cellular and whole organism level [30]. Alongside the familial cases, genome-wide association studies have revealed common genetic variants in the SNCA, LRRK2 and GBA genes which increase susceptibility to sporadic PD [29]. Animal models recapitulating these genetic mutations are an obvious choice for those looking to further scrutinise how such genetic mutations affect physiological functioning of the relevant systems in both genetic and sporadic PD cases.

## Modelling pathology

Three distinct aspects of pathology are discussed below: nigrostriatal tract degeneration, neuronal dysfunction outside the nigrostriatal tract and LB pathology/α-synucleinopathy. A checklist of the animal models that display each of these features is presented in Table 1.

**Table 1:** Summary of the pathological features seen in animal models of PD

Model	Nigrostriatal tract degeneration	Neuronal dysfunction outside of the nigrostriatal tract	Aggregates/α-synucleinopathy
MPTP	**✓**	**✓**	**✓**
6-OHDA	**✓**	**✓**	**✗**
LPS	**✓**	**✓**	**✓** ^†^
Rotenone	**✓**	**✓**	**✗**
Paraquat	**✓**	**✓**	**✓** ^†^
Permethrin	**✓**	**✓**	**✗**
Reserpine^*^	**✓**	**✓**	**ND**
Haloperidol	**✗**	**ND**	**ND**
AAV	**✓**	**✓**	**✓**
PFF	**✓**	**✓**	**✓**
Transgenic			
SNCA	**✓**	**✗**	**✓** ^†^
LRRK2	**✓**	**✓**	**Inconclusive**
GBA	**✗**	**✓**	**✗**

Ticks indicate features reportedly seen in a model, while crosses indicate features demonstrated as absent. ND, not documented; Inconclusive, literature shows evidence for and against this feature in the model. For relevant references, please see the main text. ^*^Indicates feature shown with repeated low doses of reserpine. ^†^Aggregates have been reported in addition to p129-α-synuclein.

### Nigrostriatal tract degeneration

Degeneration of the dopaminergic nigrostriatal tract is a key macroscopic pathological feature of PD and considered responsible for the cardinal motor features. This degeneration is progressive in nature. At the point of diagnosis there is estimated to be between 30 and 50% loss of tyrosine hydroxylase (TH)-positive dopaminergic neurons in the A9 region of the SNpc which results in a roughly equivalent loss of striatal dopamine innervation [31,32]. Researchers working on neurorepair mechanisms might therefore opt for a model with ‘partial’ lesion of the nigrostriatal tract, which provides scope for regeneration and sprouting of remaining neurons, while others interested in later disease states with pronounced motor symptoms may prefer a fuller lesion.

Most animal models of PD exhibit some form of degeneration of the nigrostriatal tract. The pharmacological models generated by single injection of reserpine or haloperidol are the clear exception in that these do not exhibit nigrostriatal tract degeneration. However, one group has demonstrated a reduction in striatal TH content and reduced numbers of TH^+^ cells in the SNpc following repeated injection of low doses of reserpine in mid-age (6–7-month-old) rats and mice [33,34]. These researchers have also used the model for examining neuroprotective interventions, but the partial recovery of striatal TH levels and SNpc cell counts noted 30 days after the last reserpine injection limits scope for neurorepair studies [34].

Toxin models are well-renowned for producing a sustained nigrostriatal tract degeneration that is open to examination of potential disease-modifying interventions [35,36]. Their main weakness lies in their inability to faithfully reproduce the slowly progressive nature of the degeneration; this instead happens quite rapidly in these models. One of the oldest models used in PD research, the MPTP model has been well documented to exhibit degeneration in several species including mice [37] and NHPs [38,39] when MPTP is given peripherally. Interestingly, MPTP does not induce degeneration in rats when administered peripherally [40], however, when injected directly into the SNpc, it does reduce dopaminergic cells there but without concomitant loss of striatal dopamine [41]. The dose of peripherally administered MPTP can be altered to induce the required size of lesion [42,43]. Similarly, 6-OHDA administration, at one of three sites within the nigrostriatal tract (SNpc, median forebrain bundle or striatum), induces dose-dependent nigrostriatal degeneration with loss of SNpc cells and striatal TH^+^ fibres [44]. Although effective in many animals [45,46], 6-OHDA is most commonly administered to rats or mice, as is the pesticide rotenone which induces degeneration of the TH^+^ neurons in the SNpc and loss of striatal dopamine [47]. While hampered initially by the variability of lesion size and specificity [48], optimised dosing regimens have produced more consistent lesions, leading to a renaissance in the rotenone model [49,50]. Although rotenone may be given centrally [51], it has the advantage over 6-OHDA in being effective also following systemic administration [47,49,50], ideal for those lacking stereotaxic expertise or equipment. The less commonly used paraquat model appears to produce species-dependent effects, with mice exhibiting loss of dopaminergic neurons in the SNpc in the absence of striatal dopamine loss [52]. In a study by Nasuti et al. [53], daily administration of the pesticide permethrin from postnatal day 6 to 21, led to ∼25% dopaminergic neuronal loss in the SNpc and ∼30% dopamine loss in the striatum by post-natal day 60. Although this study aged animals up to post-natal day 150 for behavioural assessment (see below), there was unfortunately no indication of whether the degeneration had progressed over this period. Finally, direct injection of the endotoxin, LPS, into the striatum [54], SNpc [55] or globus pallidus [56] of rats induced permanent degeneration of dopaminergic neurons in the SNpc. Although some groups have administered LPS peripherally [57,58], the results have been controversial: Qin et al. (2007) found that systemic LPS administration in mice induced progressive dopaminergic neuronal loss in the SNpc [57], whereas Byler et al. (2009) found no such loss with LPS alone, although LPS did exacerbate the loss induced by MPTP [58].

Nigrostriatal tract degeneration is also a key feature of the α-synuclein models of PD and in this case the pathology is much more slowly progressive in nature, better recapitulating the clinical situation. Thus, α-synuclein PFFs administered intrastriatally [22,59] or intranigrally [60] to mice and rats induced gradual and selective loss of dopaminergic neurons in the SNpc, accompanied by striatal dopamine loss over a 3–9-month period [18,22]. AAVs overexpressing wildtype or mutant α-synuclein injected into the nigrostriatal tract of rodents [19,61,62] or NHPs [21] similarly resulted in the progressive loss of dopaminergic neurons in the SNpc [63] which differed in extent based on the type, method of production and purity of the virus used.

^L444P/WT^ Transgenic mice models of PD have not been routinely successful in recapitulating dopaminergic cell loss, as extensively reviewed by others [64]. Transgenic SNCA models exhibit inconsistent outcomes with most showing little in the way of SNpc cell loss [4,65,66]. However, newer models such as the bacterial artificial chromosome (BAC)-SNCA A53T mouse do show significant, but still moderate, loss of SNpc cells [67]. GBA heterozygous mice (GBA^D409V/WT^ or GBA) are unlikely choices for examining nigrostriatal pathology given that no dopaminergic cell loss has been reported, at least up to 12 months of age [68,69]. In contrast, an age-dependent SNpc cell loss is consistently seen in LRRK2^G2019S^ transgenic mice, from 15 months of age, reaching approx. 30–40% cell loss by 24 months of age [70,71]. However, no accompanying striatal TH loss is observed, suggesting the occurrence of compensatory changes to be aware of when planning their use [70].

### Neuronal dysfunction in brain regions outside the nigrostriatal tract

In addition to SNpc degeneration, there is further widespread pathology in PD, reflected in cell dysfunction or loss in other subcortical brain regions such as the olfactory bulb, dorsal motor nucleus of the vagus (DMV), nucleus basalis of Meynert, the raphe nuclei, locus coeruleus and hypothalamus [72]. These in turn encompass disruption of additional non-dopaminergic neurotransmitter systems: cholinergic, serotonergic, noradrenergic, glutamatergic, and GABAergic. Characterisation of extra-nigral degeneration remains patchy for animal models of PD so what is included below is undoubtedly incomplete. However, given the relevance of these systems for those exploring the prodromal stage or NMS of PD, these changes may become more fully characterised in the future.

In the repeated low-dose reserpine-treated rat model, widespread reductions in TH^+^ cells are noted in regions outside the nigrostriatal tract including the locus coeruleus, prefrontal cortex and some regions of hippocampus [34]. Although not consistently seen [39], many studies show non-dopaminergic pathology caused by MPTP treatment in NHPs [38,73–76], mice [77–79] and intracranially injected rats [41]. Similarly, rotenone treatment results in a plethora of extranigral pathologies, extensively summarised elsewhere [80]. In the absence of noradrenaline transporter inhibitors which are often used to restrict 6-OHDA toxicity to dopaminergic neurons, degeneration of nearby locus coeruleus noradrenergic nuclei is seen in 6-OHDA models [81]. Additionally, 6-OHDA injections into the SNpc have been reported to increase firing patterns of neurons within the raphe nuclei [82] prefrontal cortex [83], the pedunculopontine nucleus and parafasicular nucleus of the thalamus [84].

LPS also appears to cause widespread dysfunction that varies depending on the route of administration. When administered intranasally, LPS induces changes in the olfactory bulb including TH^+^ cell reduction [85] whereas, when administered directly in the brain, it both increases TH levels and decreases choline acetyl transferase levels in the DMV [86]. LPS administered systemically leads to gradual degeneration of the noradrenergic neurons in the locus coeruleus of mice and neuronal loss in the hippocampus [87].

In the AAV–α-synuclein model the pathology is usually limited to the nigrostriatal tract, unless the AAV is injected purposefully into other brain regions, as reviewed by Koprich and colleagues [4]. The status of extranigral dysfunction has not yet been reported for PFF models, to our knowledge.

Regarding the genetic models, where reported, SNCA transgenic models do not appear to cause extra-nigral pathology, at least in neither serotonergic nor noradrenergic neurons [66]. In contrast, LRRK2^G2019S^ mice exhibit small yet significant increases in the levels of serotonin in the prefrontal cortex [70], while both dopaminergic neurons in the olfactory bulb and noradrenergic neurons in the locus coeruleus are reduced by 24 months of age [71]. Lastly, mice with the GBA^D409V/WT^ mutation develop cholinergic dysregulation in the hippocampus [69]. As already noted, it is now accepted that some of these extra-nigral dysfunctions may well underpin the non-motor features of PD and this cholinergic dysregulation in the GBA model is a prime example of such a link, given the cognitive impairment described below in this model.

### Lewy pathology/α-synucleinopathy

The microscopic pathological hallmark of PD is the presence of intracytoplasmic Lewy bodies (LBs) which harbour pathologically altered (hyperphosphorylated and aggregated) α-synuclein protein. These are often found alongside Lewy neurites, reflecting dystrophic axons. LBs also contain many other proteins such as ubiquitin, heat shock proteins and proteasomal or lysosomal proteins. According to Braak staging, LBs are first found within the olfactory bulb and DMV, then spread rostrocaudally to encompass many other subcortical nuclei, ultimately reaching the entire neocortex [88]. α-synuclein pathology has been found outside of the brain too, specifically in the spinal cord, in some sympathetic ganglia and outside of the nervous system in several peripheral organs including, but not limited to the cardiovascular system, bladder, skin and gastrointestinal (GI) system, where it has been proposed by some to originate [89]. Native α-synuclein is usually monomeric but the conformation present in LBs is a misfolded filamentous form that is prone to aggregate. Oligomeric forms of α-synuclein are now considered to be the most toxic as these are thought to act as templates for seeding and accelerating the spread of α-synuclein aggregation in the brain. α-synuclein phosphorylation at S129 (pS129 α-synuclein), as well as ubiquitination, is a common precursor to aggregation and levels of pS129 α-synuclein are often used as a surrogate for synucleinopathy in animal models.

To determine the role of α-synuclein and LBs in PD and uncover the associated pathological mechanisms, animal models that resemble this feature of the disease are required. A key criticism of toxin-based models has been their lack of expression of LB-like inclusions. That said, many toxin models do exhibit increased α-synuclein levels in brain regions such as the SNpc: paraquat-treated mice [90]; rotenone-treated [49,50] or LPS-treated [54,91] rats and mice; and MPTP-treated mice or NHPs [92,93]. More convincingly, these toxins have been shown to induce α-synuclein aggregates in dopaminergic neurons in the SNpc either transiently, in the case of paraquat [90], or more sustained in the case of MPTP [93], rotenone [49,50] and LPS [85].

As anticipated, injection of either AAVs overexpressing α-synuclein [19] or α-synuclein PFFs [94] trigger the formation of Lewy neurites and LB-like inclusions in the respective animal model. Interestingly, the PFF model shows these inclusions not only in the site of the injection (e.g. striatum) but also in other regions of the brain including the SNpc, olfactory bulb, cortex and amygdala [18]. This is an exciting observation, suggesting the PFF model could be used for investigating the spread of LB-like pathology and to identify potential ways of intervening with this key pathological process. However, it should be noted that not all studies show spreading of pathology following injection of PFFs into, for example, the gut in rats and NHPs [95].

In some SNCA transgenic models, α-synuclein-positive cytoplasmic inclusions are seen throughout the brain including the raphe magnus, brain stem, locus coeruleus and SNpc, but this is not consistently seen for all models [4,96]. Of relevance to those interested in exploring the gut–brain hypothesis of α-synuclein spread, when inclusions are seen in the gut in SNCA models, these appear to coincide with, rather than precede, brain deposition [4,96]. The picture emerging with the LRRK2^G2019S^ model is also mixed. Studies generally report no changes in pS129 α-synuclein staining at either 12 [97] or 24 [70] months of age, indicating a lack of pathology. However, the presence of higher molecular weight species of α-synuclein in the striatum and ventral midbrain in one study at 24 months of age raises the possibility of pathology [71].

Although aggregated α-synuclein is found in homozygous GBA^L444P/L444P^ Gaucher-model mice [98], both the heterozygous models (GBA^L444P/WT^ or GBA^D409V/WT^) representative of PD do not show any such aggregation [68,99], although α-synuclein levels in general may be elevated [69].

## Modelling pathogenesis

The pathogenesis of PD is complex and multifactorial and just as an individual living with PD will not exhibit all these features, neither will any single animal model. However, if delving into the mechanisms behind pathogenic features, or indeed identifying the mechanisms underpinning efficacy of a potential therapeutic is of interest, then fortunately there is at least one model for each key feature. Here, we restrict our discussion to the most established pathogenic mechanisms, many of which are interwoven: mitochondrial impairment; oxidative stress; autophagy and proteasomal dysfunction; and neuroinflammation. Table 2 presents a checklist of the animal models that reproduce each of these pathogenic features.

**Table 2:** Summary of the pathogenic features seen in animal models of PD

Model	Mitochondrial dysfunction	Oxidative stress	Autophagy and proteasomal dysfunction	Neuroinflammation
MPTP	**✓**	**✓**	**Inconclusive**	**✓**
6-OHDA	**✓**	**✓**	**✓**	**✓**
LPS	**✓**	**✓**	**✓**	**✓**
Rotenone	**✓**	**✓**	**✓**	**✓**
Paraquat	**✓**	**✓**	**✓**	**✓**
Permethrin	**✓**	**✓**	**ND**	**ND**
Reserpine	**ND**	**✓**	**ND**	**ND**
Haloperidol	**ND**	**ND**	**ND**	**ND**
AAV	**✓**	**✓**	**✓**	**✓**
PFF	**✓**	**ND**	**✓**	**✓**
Transgenic				
SNCA	**✓**	**✓**	**ND**	**ND**
LRRK2	**✓**	**✓**	**✓**	**✓**
GBA	**✓**	**✓**	**✓**	**✓**

Ticks indicate features reportedly seen in a model. ND, not documented. For relevant references, please see the main text.

### Mitochondrial dysfunction

Mitochondrial complex I activity is impaired in the SNpc in PD, supporting mitochondrial dysfunction in the pathogenesis of sporadic forms of PD. Moreover, many of the genes implicated in familial PD are associated with mitochondrial function. For example, PARK2 and PARK6 loss-of-function mutations impair the function of proteins (Parkin and PINK-1, respectively) involved in the autophagic removal of damaged mitochondria, leading to loss of this vital mitochondrial quality control process [100].

Most animal models of PD exhibit some form of mitochondrial dysfunction. In toxin models, this is hardly surprising given the toxins are known inhibitors of one or more mitochondrial complexes in the respiratory chain. Accordingly, 6-OHDA- [101], rotenone- [70,71], paraquat- and permethrin- [102–105] treated rats and MPTP-treated mice [37] display multiple features of mitochondrial dysfunction including disruption of mitochondrial complexes, impairment in oxidative phosphorylation and mitochondrial swelling [106,107]. The endotoxin, LPS, when injected directly into the striatum of rats also induces dysregulation of mitochondrial respiration [108].

α-synuclein can also accumulate inside mitochondria, leading to complex I dysfunction. Accordingly, mitochondrial fragmentation and dysfunction are observed in rats injected with AAV overexpressing A53T human α-synuclein [109] and in mice injected in the striatum with α-synuclein PPFs [110,111]. Reduced mitochondrial function and degeneration of mtDNA was similarly seen in SNCA^A53T^ transgenic mice [112]. Impaired mitochondrial function is also a feature of LRRK2^G2019S^ mice [70] and GBA^L444P/WT^ mice [99], completing what is a wide array of models for those active in this field of research and supporting the key role of mitochondrial impairment in the PD state.

### Oxidative stress

Oxidative damage in the brain in PD is evidenced by the presence of abnormal protein carbonyls, lipid peroxidation and damaged DNA bases [113]. Both an increased production of reactive oxygen species (ROS), resulting for example from the aforementioned mitochondrial impairment or from dopamine metabolism via monoamine oxidase B, and a reduced level of antioxidant defences (reduced glutathione) combine to drive oxidative stress in PD. Given the ubiquitous nature of mitochondrial impairment in animal models of PD, it is unsurprising that the generation of ROS or reactive nitrogen species (RNS), supporting oxidative or nitrergic stress is a common feature of many models.

In the case of the pharmacological PD models induced by low-dose reserpine, oxidative damage was observed in the striatum in both rat and mouse models [11,34]. All toxin models exhibit oxidative stress due to their proposed mechanism of action; this has been well-reviewed previously [2] with rotenone shown elsewhere to follow suit [114]. When LPS is injected directly into the brain a rise in iNOS expression occurs, triggering production primarily of RNS alongside ROS [54]. In relation to the α-synuclein models, rats treated with AAV overexpressing human A53T α-synuclein display clear oxidative stress [109], while the PFF model again displays elevated iNOS, supportive of RNS, but only when the injected PFFs are phosphorylated [115]. Finally, many of the genetic models of PD including SNCA and LRRK2 also exhibit oxidative stress, as extensively reviewed by Varçin and colleagues [116], while reports of increased ROS production have also been seen in the GBA transgenic mouse models [68,99].

It is important to highlight that many of the above studies demonstrate ROS production in neurons from these animals *ex vivo*, due to the technical difficulties in assessing these parameters *in vivo*.

### Autophagy and proteasomal dysfunction

Damaged proteins such as those affected by oxidative stress, or misfolded α-synuclein, are cleared by two main systems: the ubiquitin–proteasome system (UPS) and the autophagy lysosome system. The former involves the labelling of damaged proteins with ubiquitin prior to their transportation to the proteasome for degradation [117]. In idiopathic PD brain, reduced catalytic activity of the proteasome system is present, alongside reduced expression of 20S proteasome α-subunit [118]. In familial cases, loss of function mutations in the PARK2 gene (resulting in reduced E3 ubiquitin ligase) and in the PARK5 gene (resulting in reduced ubiquitin C-terminal hydrolase) also result in a compromised UPS [100]. Three different forms of autophagy exist: microautophagy, in which the lysosome itself engulfs damaged proteins; macroautophagy in which the autophagosome engulfs these prior to fusing with the lysosome; and chaperone-mediated autophagy in which molecular chaperones target and transport damaged proteins to the lysosome [119]. Reduced levels of lysosomal proteins (e.g. LAMP1 and LAMP2A) or molecular chaperones such as certain heat-shock family proteins support autophagy dysfunction in idiopathic PD [120], while mutations in genes such as GBA1 which reduces activity of the lysosomal enzyme GBA [121] and PARK2 or PARK6 which are known to impair autophagy [100] support the involvement of the autophagy lysosomal system in the pathogenesis of familial PD case.

Proteasome dysfunction was modelled early on in rodents using specific proteasome inhibitors [122]. However, use of these inhibitors to generate PD models is no longer favoured after reports of a lack of reproducibility in various species [123]. Although MPTP-treated mice exhibit proteasome dysfunction, as indicated by the 20S proteasome activity assay [35], proteasome inhibitors do not enhance MPTP neurotoxicity [124], but rather offer neuroprotection [124–126], suggesting this model is not ideal for those wishing to model proteasome dysfunction in PD.

Autophagy dysfunction, on the other hand, is consistently observed in a wide variety of existing models. For example, of the toxin models, impairment of autophagy flux has been detected in the 6-OHDA rat model, where LAMP1 expression and lysosomal protease activity were reduced [127] and in the rotenone rat model, in which the impairment was associated with the formation of autophagic vacuoles [107]. LPS administered peripherally in mice also impairs autophagic function, as indicated by abnormal expression of autophagic markers such as LC3-II and HDAC-6 [128]. Through detailed *in vitro* studies, Grassi and colleagues have suggested that mice injected with α-synuclein PFFs that exhibit deposition of a toxic species of α-synuclein do so due to impaired autophagy [111], but a direct demonstration of reduced autophagy *in vivo* remains to be seen. However, rats injected with AAV–α-synuclein in the olfactory bulb exhibit increased expression of autophagic markers [129], while injection into the SN has been shown to result in impairment of the UPS [130]. Among the transgenic models under consideration here, GBA^L444P/WT^ mice are believed to express impaired mitophagy secondary to impaired mitochondrial function and reduced autophagy–lysosomal degradation efficacy [99]. In LRRK2^G2019S^ mice, impaired autophagy is also implicated, this time by the accumulation of autophagic vacuoles in both the striatum and cortex [70].

### Neuroinflammation

Neuroinflammation is evident in the brain in PD and is believed by most to contribute to the pathogenesis. This is evidenced by the occurrence of astrocytosis, microgliosis, infiltration of immune cells, complement activation and increased production of pro-inflammatory cytokines such as IL-1β and TNF-α in the brain in PD, extensively reviewed elsewhere [131]. Data from PET imaging studies using the microglial marker [^11^C]-PK11195 further support microglial activation as an early feature in PD in many brain regions including the brainstem, SN, striatum and frontotemporal cortices [132]. Given that neuroinflammation is a hallmark of PD, animals models that replicate this feature provide a crucial tool to investigate the underlying mechanisms and potential insights into new targets for therapeutics. Neuroinflammation is present in most animal models of PD, providing interested researchers with many choices.

Activation of microglia and changes in inflammatory signals have been reported in both mice and NHPs after MPTP treatment [133–136], while astrogliosis (increased GFAP) and microgliosis (increased Iba1) are well-documented in the SNpc and striatum of 6-OHDA-treated mice and rats [137,138] and rotenone-treated rats [139,140]. McCormack and colleagues have shown that mice treated with paraquat also exhibit increased inflammatory signals [141], however, few studies have investigated whether they impact on microglial morphology or function [142,143]. LPS, which is known to cause an inflammatory response via binding to the TLR4, increases levels of proinflammatory cytokines when administered directly into the SNpc to model PD [144].

Regarding the α-synuclein models, following AAV–α-synuclein injection an increase in microglial number and proinflammatory cytokines is seen in the striatum prior to the dopaminergic neuronal death [145]. Moreover, studies in the PFF model have shown alterations in the levels of immune cells in the periphery [59], microgliosis in several brain regions including the SNpc and striatum and the presence of reactive astrocytes in the SNpc [18,111,146] all supportive of neuroinflammation occurrence.

Neuroinflammation is less studied in the genetic models and to date SNCA transgenic mice show a very mixed response [4]. This mixed pattern is followed with other models too. For example, in 24-month-old LRRK2^G2019S^ mice, increased GFAP immunoreactivity is seen in the SNpc and striatum, but without changes in microglial number [71]. In contrast, in GBA^D409V/WT^ mice, a heightened inflammatory response has been reported in the hippocampus [69] but was absent from the SNpc [88]. In general, more support is needed before the utility of the genetic models for the study of neuroinflammation can be established.

## Modelling the motor symptoms

Even though PD is increasingly recognised as a syndrome harbouring both motor and non-motor features, its diagnosis is still reliant on the motor deficits, indicating the importance of these in this life-changing condition. A key motor symptom of PD is bradykinesia. Tremor is another cardinal symptom of PD, alongside rigidity and postural imbalance. For the purposes of this review, a detailed explanation of the different clinical features is not warranted but the reader can explore more in either of these comprehensive reviews [147,148]. Here, we consider the models that display motor symptoms and highlight those in which symptoms are reversed by L-DOPA, as summarised in Table 3.

**Table 3:** Summary of the motor features seen in animal models of PD

Model	Motor deficits	Responds to L-DOPA	Develops L-DOPA-induced dyskinesias
MPTP	**✓**	**✓**	**✓**
6-OHDA	**✓**	**✓**	**✓**
LPS	**✓**	**ND**	**ND**
Rotenone	**✓**	**✓**	**✓**
Paraquat	**✓**	**✓**	**ND**
Permethrin	**✓**	**✓**	**ND**
Reserpine	**✓**	**✓**	**ND**
Haloperidol	**✓**	**ND**	**ND**
AAV	**✓**	**Inconclusive**	**ND**
PFF	**✓**	**ND**	**ND**
Transgenic			
SNCA	**Inconclusive**	**ND**	**ND**
LRRK2	**✓**	**✓**	**ND**
GBA	**✗**	**n/a**	**ND**

Ticks indicate features reportedly seen in a model, while cross indicates features demonstrated as absent. ND, not documented; Inconclusive, literature shows evidence for and against this feature in the model; n/a, not applicable, due to the lack of motor deficits. For relevant references, please see the main text.

The rapid pharmacological models of PD, generated by haloperidol or reserpine, have been successful at reproducing some of the motor signs of PD including abnormal posturing (catalepsy), bradykinesia and limb or core rigidity [10–12]. As such these acute models offer relatively simple and rapid turnaround platforms for testing the acute effects of novel symptomatic treatments [149]. In the repeated low-dose reserpine models, rats and mice display progressive motor impairment and although this reverses within 30 days of the last injection, it offers a greater window for behavioural assessment [33,34,150].

Most of the toxin-based models display a range of motor signs that can be assessed at intervals as required. Where toxins like 6-OHDA or rotenone are administered unilaterally, the classic contra- or ipsi-versive rotational responses to apomorphine and amphetamine, respectively, can be measured. These are largely used to indicate the extent of the lesion so can offer insight into the efficacy of a protective or restorative drug treatment, for example, or be used to assign animals with similar-sized predicted lesions into matched treatment groups. Other readouts of motor impairment in these animals include contralateral paw akinesia, which can be measured using adjusted step, cylinder or foot slip tests [36,44,151]. Permethrin treatment in rats also causes asymmetric posture, foot slips and dragging of hindlimbs using the beam walking test [53]. Bilateral lesions, on the other hand, as usually generated in MPTP-treated mice or systemically administered rotenone models, may cause an overall reduction in locomotor activity, or bradykinesia, evident in locomotor arenas or using rotarod, with additional abnormalities in rearing and postural instability noted with rotenone treatment in rats [80]. Likewise, paraquat treatment in mice induces a reduction in locomotor activity and rearing [52]. The most naturalistic group of motor symptoms are of course seen in the MPTP-treated NHP models of PD which display bradykinesia coupled with rigidity and tremor [152,153].

Similar motor impairments induced in the α-synuclein models, including those generated by intranigral AAV overexpression of α-synuclein [61] or by unilateral injection of α-synuclein PFFs [18,94], are notably more progressive in nature. Indeed, in the PFF models, motor impairment is not often evident until 9-months post PFF injection rendering these models significantly more expensive to run and probably not, therefore, the first choice for simple symptomatic interventions but rather for monitoring the functional impact of potential disease modifying therapies.

Genetic models of PD are also not recommended if monitoring motor dysfunction is a key study aim. Nevertheless, some of these do express motor impairment over time. For example, LRRK2 mice develop gait and coordination issues, usually by 24 months of age [71,154], whereas the SNCA^A53T^ mice develop significant bradykinesia and hypokinesia in some, but not all studies [4,96,155,156]. GBA heterozygous mouse models (GBA^D409V/WT^, GBA^L444P/WT^) on the other hand, appear to consistently exhibit no changes in motor function [68,69].

Many of these behavioural perturbations are ameliorated by treatment with L-DOPA, showing a good predictive validity of the models for motor symptom-relieving drugs. This is the case for reserpine-induced akinesia [157,158] and the kinetic or postural dysfunctions manifested in the 6-OHDA [159,160], rotenone [161,162], MPTP and permethrin toxin models [2,163–165]. Moreover, L-DOPA improves performance of the LRRK2 mouse in the cylinder, open field [166] and pole test [71].

## Modelling L-DOPA-induced dyskinesia

Long-term use with L-DOPA in PD patients can evoke disabling involuntary uncontrolled chorea-like movements or dystonias, collectively termed L-DOPA-induced dyskinesias (LIDs) [167,168]. The mechanisms behind these LIDs remain to be fully elucidated, but aberrant glutamate signalling is implicated [169], and supported by the weak NMDA receptor antagonist amantadine being one of few drugs clinically used in the fight against LID [170]. To further increase our understanding of LID and aid in discovering anti-dyskinetic therapeutics, animal models of PD that exhibit LID are available for use. MPTP-treated NHPs (common marmosets and macaques) were the first animal models used to recapitulate LID, displaying naturalistic choreic and dystonic movements recapitulating those seen in patients following chronic L-DOPA administration [171–174]. As extensively reviewed by Cenci and Crossman [175], it was subsequently realised that the 6-OHDA toxin models in both rats and mice readily developed abnormal involuntary movements, akin to LIDs, within 10–14 days of L-DOPA treatment. These rodent models of LID vastly increase accessibility for more researchers to investigate this troublesome side effect of L-DOPA and, like their NHP counterparts, have been validated in that their LIDs may be reversed by amantadine [176,177]. Albeit less-well studied, rotenone-treated rats have also been shown to develop LIDs [178,179] so would be worth considering in future dyskinesia research studies.

## Modelling the NMS

Despite being identified in the very first description of PD by James Parkinson [180], NMS are a feature of the condition that has been somewhat overlooked in comparison to the motor features. This situation has changed recently, and NMS are starting to receive the attention they deserve given their significant impact on the quality of life of people living with Parkinson’s [181,182]. Identifying animal models that can replicate some NMS is a crucial step in gaining insight into the mechanisms involved in their manifestation and to provide a platform for the testing of novel therapeutic interventions.

Due to the extensive literature investigating potential molecular and pathological aspects of NMS, this section will focus on models that exhibit NMS with tangible behavioural readouts in animals: somnolescent alterations (sleep and fatigue); sensory abnormalities (anosmia and pain manifestation); cognitive deficits; psychiatric changes (affective, anxiotypic or psychomimetic); and organ system dysfunction (GI system and bladder). A summary of the animal models expressing these NMS is given in Table 4.

**Table 4:** Summary the non-motor features seen in animal models of PD

Model	Sensory abnormalities	Somnolescent alterations	Cognitive deficits	Psychiatric changes	Organ system dysfunction
MPTP	**✓**	**✓**	**✓**	**✓**	**✓**
6-OHDA	**✓**	**✓**	**✓**	**✓**	**✓**
LPS	**ND**	**ND**	**✓**	**✓**	**✓**
Rotenone	**✓**	**✓**	**✓**	**✓**	**✓**
Paraquat	**✓**	**ND**	**✓**	**✓**	**✓**
Permethrin	**ND**	**ND**	**✓**	**✓**	**✓**
Reserpine	**✓**	**ND**	**✓**	**✓**	**ND**
Haloperidol	**✗**	**ND**	**ND**	**ND**	**ND**
AAV	**✓**	**ND**	**ND**	**✓**	**ND**
PFF	**✓**	**✓**	**✓**	**✓**	**✓**
Transgenic					
SNCA	**✓**	**✓**	**✓**	**✗**	**✓**
LRRK2	**✓**	**✓**	**✓**	**Inconclusive**	**✓**
GBA	**✗**	**ND**	**✓**	**ND**	**✗**

Ticks indicate features reportedly seen in a model, while crosses indicate features demonstrated as absent. ND, not documented; Inconclusive, literature shows evidence for and against this feature in the model. For relevant references, please see the main text.

### Somnolescent alterations

People with PD often have an increased occurrence of feeling fatigued and also develop REM sleep behaviour disorder [183,184] where they act out dreams (often violently) during the prodromal stages of the disease. Due to the subjective nature of fatigue, it is difficult to isolate and quantify it using an experimental behavioural paradigm. Therefore, literature on PD models that exhibit fatigue is severely lacking. However, alterations in sleep patterns and movement during sleep can be assessed *in vivo* as an index of somnolescent alterations.

This aspect of PD has been successfully measured in toxin models. Telemetry implants in MPTP-treated NHPs show deregulation of REM sleep and increased daytime sleepiness prior to the classic MPTP-induced motor deficits [185,186]. This timeline is consistent with the prodromal appearance of these symptoms in PD. Similar effects can be seen in rodents, with either a unilateral 6-OHDA lesion or rotenone treatment [187,188] in which sleep patterns are disrupted by increasing REM epochs. While alterations in sleep behaviour have been seen following administration of α-synuclein PFFs, this only occurs when fibrils are injected into the laterodorsal tegmental nucleus [189], not when injected into the striatum as is typical for the PFF model of PD. Of the transgenic models, BAC-SNCA A53T mice display REM sleep without atonia, which is a key feature of REM sleep behaviour disorder, at as early as 5 months of age [67]. LRRK2^G2019S^ mice also display sleep problems, manifest as increased sleep fragmentation that reduces sleep quality [190]. However, despite the clear link between GBA mutations and REM sleep behaviour disorder in patients [191], there are no reports to our knowledge of altered sleep patterns in GBA mice.

### Sensory abnormalities

Similar to the somnolescent changes that occur in PD, sensory abnormalities also start prior to the motor symptoms. One of the key early-stage symptoms is a loss of sense of smell (anosmia). The prevalence of anosmia has been reported in up to 90% of people with PD. This is thought to occur due to synuclein pathology in the olfactory bulb at the first stages of PD [88]. In animals, anosmia is represented as a failure to discriminate between odours.

The repeated low-dose reserpine mouse model has been shown to display olfactory impairment, failing to discriminate between familiar and non-familiar odour cues [33]. With regard to the toxin models, MPTP, 6-OHDA, paraquat and rotenone models in rodents or NHPs all exhibit reductions in olfactory acuity in scent discrimination paradigms [36,102,185,192–195]. The impact of synucleinopathies on olfaction have been recently highlighted by the deficits seen when AAV–α-synuclein is injected into the olfactory bulb [118]. This link is further supported by studies reporting olfactory impairment following the injection of PFFs into the sublaterodorsal tegmental nucleus, gut and olfactory bulb [168,175,176]. However, whether olfaction is affected in the striatally injected PFF model of PD is not yet known. A lack of odour discrimination has also been observed in a number of the SNCA genetic models by 5 months of age, well before any motor impairment is seen [67,196,197]. While also evident in LRRK2^R1441C^ mice [154], this olfactory impairment is only manifested at 24 months, alongside the motor impairment [198]. To our knowledge, GBA mice models of PD have not yet been assessed for olfactory function.

Another common sensory change that occurs in PD is the expression of pain, with a prevalence of up to 80%. This normally manifests as spontaneous pain (musculoskeletal, nocturnal, dystonic, central and neuropathic) and noxious hypersensitivity (reduced thresholds to painful stimuli) [199]. Buhidma and colleagues have recently extensively reviewed the animal models of pain in PD [200]. Although a few individual studies have investigated nociceptive threshold changes in MPTP, reserpine and LRRK2 models [201–203], findings are not always consistent [198]. The most well-characterised and consistent model for pain in PD is the 6-OHDA toxin model in rodents [200]. Briefly, a 6-OHDA unilateral lesion anywhere in the nigrostriatal tract causes bilateral hypersensitivity in a variety of nociceptive modalities. This altered nociceptive processing supports use of the 6-OHDA rodent model as a platform for novel analgesic testing. No reports of nociceptive testing appear to be published yet for GBA or SNCA genetic models.

### Cognitive deficits

Unlike many NMS in PD, dementia is a common symptom that manifests at the later stages of the disease. This is thought to occur when the LB pathology spreads to and causes dysfunction in the cortical and hippocampal regions of the brain that govern memory, executive and higher thinking. This NMS is recapitulated in a surprisingly large number of the animal models of PD.

The repeated low-dose reserpine model in both mice and rats exhibits cognitive impairment, evidenced by a lack of novel object recognition [33,34]. A lot of the early pivotal studies using MPTP in NHPs showed that exposure to this toxin impacts cognitive function [143,185,203–206]. Since then, rodent studies have reported similar deficits with MPTP as well as other toxins (paraquat, permethrin, 6-OHDA, rotenone and LPS) [53,114,207–209].

Reduced cognitive function is also seen in mice following the injection of α-synuclein PFFs into the gut of mice [210]. Although this was concurrent with motor dysfunction, which does not mirror what is seen in many PD cases, these findings support the idea that dementia may result from the upward spread of synuclein pathology from the gut into the brain.

The picture with the genetic models is promising but inconsistent. In some studies, LRRK2^R1441G^ mice show no cognitive deficits at 21 months compared with aged-matched w/t controls [198]. However, in other studies using the LRRK2^G2019S^ model, a worsening in cognitive performance occurred in 6, 12 and 18 months old mice [211] with spatial memory deficit evident at 10 months of age [212]. In support of the positive outcome studies, given the gain-of-function nature of this mutation, mice overexpressing LRRK2 also displayed cognitive deficits at 12 months of age [213].

As noted above, heterozygous GBA mice exhibit hippocampal changes, so it is not surprising these mice express cognitive deficits. Indeed, deficits are seen with this model in an array of cognitive paradigms but, when deciding whether to select this model for a given study, it should be remembered that these impairments occur in the absence of either motor symptoms or nigrostriatal degeneration [69,98]. Finally, there is evidence for reduced social recognition and other cognitive deficits in SNCA overexpressing mice [214] or, as extensively reviewed, in several SNCA transgenic models with differing times of onset apparent in each [215].

### Psychiatric changes

In the later stages of the prodromal phase of PD, prior to motor impairment, diagnoses of anxiety and depression are increased. These symptoms tend to be unresponsive to commonly used antidepressants and anxiolytics. Many different animal models of PD exhibit behaviours representative of anxiety or depression. Given that repeated low doses of reserpine have been used to model depression-like behaviour *per se*, it is no surprise that this dosing paradigm causes increased immobility and reductions in swimming time in the forced swim test [150]. When administered in this way to specifically induce the PD model, reserpine-treated mice also display anxiety-like behaviour in the elevated plus maze [33].

Models induced by MPTP, 6-OHDA and LPS treatment in rodents express similar anxiety-like symptoms [41,78,203,209], while the rotenone model only shows depressive behaviour [41,216]. Permethrin on the other hand fails to exhibit anxiety-like behaviour [207] and paraquat has only been shown to date to cause depression- and anxiety-like behaviour when given alongside maneb, a type of fungicide [217].

When AAV–α-synuclein is injected into the SNpc, it too causes depressive behaviour although this occurs prior to motor deficits, unlike the clinical situation [218]. Mice also exhibit mood changes when α-synuclein PFFs are injected into the sublaterodorsal tegmental nucleus [189]. Interestingly, when PFFs are injected into the gut, mice develop anxiety- and depression-like behaviour, again supporting the spread of pathology along the gut–brain axis [210].

Evidence of psychiatric symptoms in the genetic models is relatively sparse. However, contrary to the clinical situation, SNCA mice generally show lower, rather than increased levels of anxiety in the tail suspension and open field tests [219]. LRRK2 mice, on the other hand, show conflicting effects depending on the mutation. Thus while anxiety- and depression-type behaviours are evident between 10 and 12 months of age, prior to any motor dysfunction, in LRRK2^G2019S^ mice [220], there is either no manifestation, or reduced levels of anxiety and depression-like behaviours in LRRK2^R1441C/G^ mice [154,198]. No reports exist for the GBA heterozygous mouse model of PD.

In addition to anxiety and depression, people with PD tend to develop psychosis and hallucinations, often because of long-term exposure to dopamine-based therapeutics that treat the motor symptoms. Typically, psychotomimetic behaviour is difficult to assess *in vivo*, however one study has shown a range of psychosis-like behaviour in MPTP-treated NHPs. Specifically, MPTP-treated marmosets modelling the advanced stages of PD displayed a range of behaviours indicative of psychosis when on L-DOPA (e.g., agitation, tracking non-existent stimuli and stereotyped movements). Moreover, these behaviours were reversible with the atypical anti-psychotics, clozapine and quetiapine [221]. Although studies elsewhere are limited, it is worth noting that pre-pulse inhibition of the acoustic startle reflex, a measure of sensorimotor gating and psychotic indicator was not affected at 6 or 15 months of age in either of the LRRK2 models [70].

### Organ system dysfunction

GI dysfunction is increasingly considered the vanguard symptom of PD as it often predates any of the other symptoms by many years. Whether this is due to central PD pathology spreading down to the gut or whether PD pathology begins in the gut is an exciting hypothesis still being debated [222–225], but outside of the scope of this review. Here we focus simply on whether GI dysfunction is seen in the animal models. Although toxin models do not recapitulate a spreading of pathology, central injections of 6-OHDA do delay gastric emptying 1-week post-lesion [226], indicating GI dysfunction. GI dysfunction is also seen in rodent models induced by peripheral administration of MPTP, paraquat and rotenone [203,227–230], while peripheral injections of LPS also alters GI tract permeability without any evidence of nigral dopamine-loss [231]. In all of these cases the underlying cause of the GI dysfunction remains to be established. Not surprisingly, injection of α-synuclein PFFs into the gut induces GI dysmotility, but whether this occurs following intrastriatal PFF injection is not known [210]. Finally, of the genetic models, GI dysfunction is reported in LRRK2^R1441G^ mice, where it occurs as early as 6 months of age, well before the nigrostriatal tract pathology [198].

Among the other organs that are often dysfunctional in PD is the lower urinary tract. Symptoms, such as increased urinary frequency, nocturia, and urinary incontinence, develop with a high frequency (27–63.9% of all patients). This feature is seen in a number of the animal models of PD. For example, in the MPTP-treated NHP model, lower bladder volumes and reduced pressure thresholds for inducing the micturition reflex are seen [232,233]. Similarly, the 6-OHDA rat model which has been heavily investigated, exhibits reduced bladder capacity, lower thresholds for the micturition reflex, as well as bladder overactivity [234–240]. The final model to highlight for those interested in bladder dysfunction is the SNCA^A53T^ mouse model which has more frequent contractions of the bladder and smaller bladder voiding volumes prior to any motor impairment [241].

Although less commonly affected, the final organ system considered here is the cardiovascular system. Alterations in the cardiovascular system in PD normally manifest as cardiac autonomic dysfunction, cardiomyopathy, coronary heart disease, arrhythmias, conduction defects and sudden cardiac death [242,243]. Changes in cardiovascular control are seen in many models, though not always of an equivalent nature. In MPTP-treated mice, for example, long lasting baroreflex desensitisation, tachycardia, and decreased heart rate variability are seen compared with shams [244]. Similar issues are seen post-permethrin treatment in rats [245]. In 6-OHDA-lesioned rats, on the other hand, while decreased systemic arterial pressure variation is noted, a combination of reduced heart rate and mean arterial pressure plus impaired compensation in a postural hypotension test indicate impaired autonomic function especially of the sympathetic system [246–248]. These changes occur without any alterations in the ECG [249]. Using telemetry devices, Griffioen and colleagues, demonstrated, as seen in MPTP mice, elevated heart rate in SNCA^A53T^ mice alongside impaired autonomic control [250]. No studies have yet reported cardiovascular impairment in LRRK2 mice, however this is unlikely to be observed given the much-reduced incidence of cardiovascular complications reported in PD patients carrying LRRK2^R1441G / G2019S^ mutations compared with sporadic cases [251]. Similarly, no reports are published relating to cardiovascular function in either GBA^L444P/WT^ or GBA^D409V/WT^ mice.

## Conclusions

**Figure 1:**
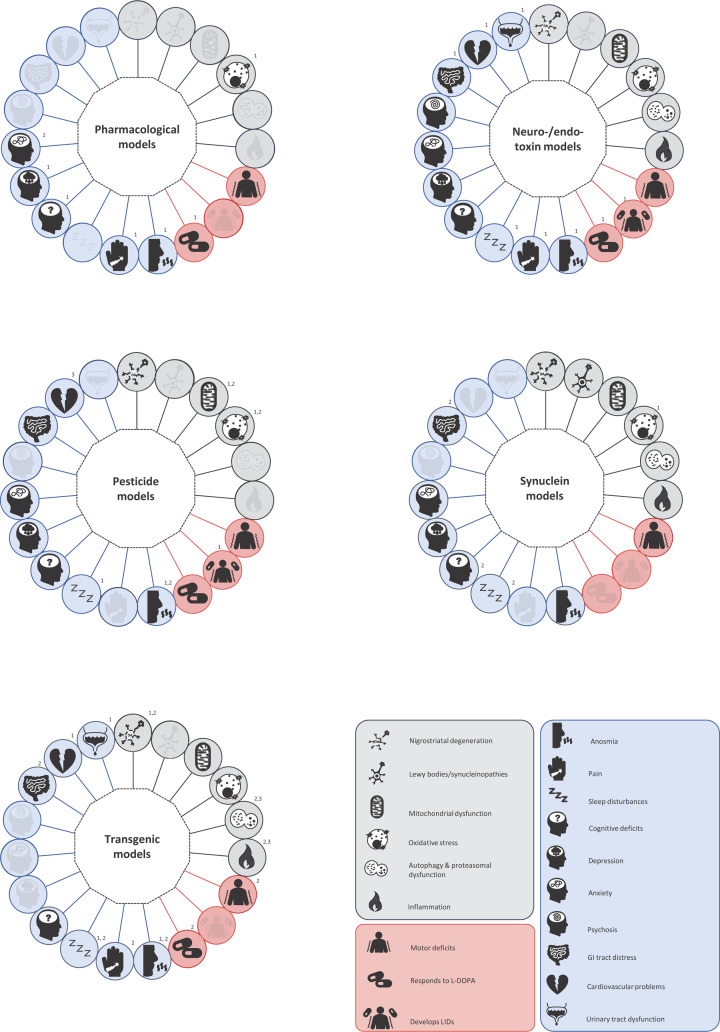
Graphical summary of the key pathological, pathogenic and symptomatic features expressed for the different classes of animal model of PD For clarity, all features considered in this review are represented around each model class, but only those expressed are not greyed out. When used, numbers are shown on the top right of the circle they are associated with. (a) Pharmacological models; 1 applies only to the reserpine model. (b) Neuro-/endo-toxin models; 1 applies only to the neurotoxin models. (c) Pesticide toxin models; 1 applies only to the rotenone model, 2 applies only to the paraquat model. (d) α-Synuclein models; 1 applies only to the α-synuclein–AAV model, 2 applies only to the PFF model. (e) Genetic models; 1 applies only to SNCA models. 2 applies only to LRRK2 models. 3 applies only to GBA models.

As summarised in Figure 1, it is clear that researchers in the PD field have a wide array of animal models at their disposal and that many of these recapitulate multiple features of the disease from pathology and pathogenesis to the motor and non-motor symptoms. However, this extensive choice can make selecting the model for a given study a daunting prospect, especially so for one that aims to address multiple aspects of the disease simultaneously. By compiling this review, we set out to support this decision-making process. We have provided researchers with a snapshot of which models express each of the key features of PD (Tables 1-4), providing further details and some supporting references within the text. Conversely, Figure 1 presents a more visual illustration, this time summarising which features of PD a given class of animal models can replicate. This figure will be especially informative for studies that demand a model which displays a specific combination of features. Armed with this information, we hope that both experienced researchers and those new to the field of PD research will feel more confident when selecting the optimal animal model for their study needs.

## Data Availability

It is not relevant as it is a review article.

## Abbreviations

AAV: Adeno-associated viral vector
BAC: bacterial artificial chromosome
BSSG: β-sitosterol d-glucoside
DMV: dorsal motor nucleus of the vagus
GBA: glucocerebrosidase
GI: gastrointestinal
iNOS: inducible nitric oxide synthase
LB: Lewy body
LID: L-DOPA-induced dyskinesia
LRRK2: leucine-rich repeat kinase
LPS: lipopolysaccharide
L-DOPA: levodopa
MPTP: 1-methyl-4-phenyl-1,2,3,6-tetrahydropyridine
NHP: non-human primate
NMDA: N-methyl-D-aspartate
NMS: non-motor symptoms
PD: Parkinson’s disease
PFF: pre-formed fibril
REM: rapid eye movement
RNS: reactive nitrogen species
ROS: reactive oxygen species
SNCA: α-synuclein
SNpc: substantia nigra pars compacta
UPS: ubiquitin–proteasome system
6-OHDA: 6-hydroxydopamine
